# Extensive surveillance of mosquitoes and molecular investigation of arboviruses in Central Iran

**DOI:** 10.1097/MS9.0000000000002826

**Published:** 2025-01-09

**Authors:** Fatemeh Abedi-Astaneh, Hedaiatollah R. Rad, Hassan Izanlou, Seied A. Hosseinalipour, Amir Hamta, Mohammad Eshaghieh, Mahdi Ebrahimi, Mohammad A. Ansari-Cheshmeh, Mohammad H. Pouriayevali, Mostafa Salehi-Vaziri, Tahmineh Jalali, Asghar Talbalaghi, Ebrahim Abbasi

**Affiliations:** a Research Centre for Environmental Pollutions, Deputy for Health, Qom University of Medical Sciences, Qom, Iran; b Department of Biology and Control of Disease Vectors, School of Health, Shiraz University of Medical Sciences, Shiraz, Iran; c Department of Arboviruses and Viral Hemorrhagic Fevers (National Reference Laboratory), Pasteur Institute of Iran, Tehran, Iran

**Keywords:** arboviruses, Culicidae, entomological surveillance, Iran, PCR, vector

## Abstract

**Background:**

Arboviruses are one of the greatest threats to animal and public health. *Culicidae* family is one of the most important vectors for the transmission of arboviruses in the world. According to the geographical, demographic, and climatic features of Qom city in Iran, it can be a suitable region for vectors and therefore transmission of arboviruses.

**Methods:**

In this study, which was conducted between 2019 and 2020 in different parts of Qom city, 83 414 mosquitoes were collected, and after evaluating the species of mosquitoes based on morphological and molecular detection, the presence of alphaviruses, flaviviruses, and phleboviruses were evaluated using genus-specific Reverse transcription polymerase chain reaction (RT-PCR) assays.

**Results:**

In this study, *Culex tarsalis, Culex theileri, and Culex quinquefasciatus* were detected, including the first recorded presence of *Culex tarsalis* in Iran. No infections with alphavirus, flavivirus, or phlebovirus were identified in the collected mosquitoes.

**Conclusion:**

Climatic and weather changes are the basis for the growth and spread of vectors and, consequently, the spread of arboviral diseases, and this issue seems to be very important to the necessity of increasing and continuing entomological and virological studies.

## Introduction

Arthropod-borne viruses circulate between vertebrate hosts and arthropod vectors. Arboviruses has been considered a serious public health challenge as they make up more than 17% of infections and have affected the lives of millions of people in a wide geographical area around the world^[1]^. It is estimated that more than 520 viruses are transmitted by mosquitoes of which 130 are medically important^[2,3]^. The most important arboviruses mainly belong to the families of *Togaviridae* (genus Alphavirus), *Flaviridae* (genus Flavivirus), Orthobunyavirus, and Phlebovirus^[4,5]^. Mosquitoes such as *Culicidae* family are well-known vectors for arboviruses worldwide^[6]^. There are 3100 species, 34 genera, and three subfamilies in the *Culicidae* family; The genuses/genera of the two subfamilies Anophelinae (*Anopheles*) and Culicinae (*Aedes, Culex, Culiseta, Mansonia, Hemagogus, Sabethes,* and *psoraphora*), are the most important vectors of arboviruses^[7]^.

In recent years, the risk of spreading mosquito vectors of arboviruses has increased significantly due to climate change and globalization, so that in the last decade, the world has seen widespread outbreaks and epidemics of these viruses, such as chikungunya, West Nile, Zika, and dengue viruses^[8]^. Apart from Went Nile virus, there have been no reports of autochthonous transmission of Zika, dengue, and chikungunya viruses^[9]^. However, imported cases of chikungunya and dengue viruses have been documented from endemic countries mostly in East Asia^[10,11]^.

The study addresses the presence of *Culex tarsalis* in Iran, linking it to similar research in Southern Europe and the Middle East, where climatic conditions foster mosquito breeding and arbovirus transmission. It highlights the shared Mediterranean climate of countries like Spain and Italy, where *C. tarsalis* is associated with West Nile virus outbreaks. The findings emphasize the need for expanded surveillance in Iran and neighboring regions, noting that changing climates and human activities might encourage the spread of this species. The study enriches existing knowledge by documenting a previously unreported mosquito species in Iran and underscores the importance of monitoring emerging vectors in light of global climate change and urbanization, which may elevate arbovirus transmission risks^[12,13]^.

Owing to the fact that there is a close association between arboviruses and their mosquito vectors, the geographical distribution of vectors can be used to estimate the risk of emergence of arboviral infections and their outbreaks^[14]^. Although several studies investigated the mosquitoes’ fauna in Iran^[15-17]^, there is no information regarding the arboviruses’ vectors fauna in Qom, Central Iran. The Qom is the main crossroad of Iran for the transportation of goods and passengers of Iran. Qom is the pole of Shiism in the world; it has holy places such as the holy shrine of Bibi Fatima Masoumeh, the holy mosque of Jamkaran; and the diversity of the presence of immigrants and pilgrims with ethnicities from most parts of Iran and nationalists from more than 100 countries. This city is the transportation crossroad of the country, so it is very important to control infectious diseases, carriers, and disease reservoirs.

Since some Aedes species are highly adaptable to various climatic, geographical, and nutritional conditions, they have successfully spread across tropical, subtropical, and temperate regions. Additionally, as urban insects, they are often transported by human vehicles. Notably, vehicles carrying pilgrims and travelers from Iran’s southeastern neighboring countries sometimes pass-through border provinces before reaching Qom, while others arrive directly from these countries. Therefore, it is possible to transfer Aedes mosquitoes of these vehicles to the city of Qom—the place of residence for a few days of the these people. The risk of spreading mosquito vectors of arboviruses has increased significantly due to climate change and globalization, so that in the last decade, the world has seen widespread outbreaks and epidemics of these viruses, such as dengue, Zika, chikungunya, and West Nile viruses. Because the exact identification of mosquitoes is essential for controlling the species that are vectors of human and animal diseases^[18]^. Therefore, this study was designed to discover the circulating mosquitoes in Qom province and investigate the alphaviruses, flaviviruses, and phleboviruses infections in them.

The detection of *C. tarsalis* in Iran, despite the absence of arboviruses in this study, is significant for public health planning and vector control efforts. This species, which could become a vector for viruses like West Nile, poses a potential future disease risk as environmental conditions change. Early identification of *C. tarsalis* allows for proactive surveillance and control strategies to prevent disease outbreaks. Additionally, as Iran borders countries with prevalent arboviruses, awareness of cross-border transmission risks is crucial. The study underscores the importance of enhancing vector surveillance and management in response to emerging mosquito species^[19-21]^.

We will address this by discussing possible reasons for negative arbovirus findings, including seasonal timing, assay sensitivity, and low prevalence of arboviruses in the region. These factors likely contributed to the lack of detected viruses, despite thorough surveillance. This discussion will highlight the importance of continuous monitoring to record any seasonal or sporadic changes in arbovirus circulation.

Arbovirus surveillance is increasingly important for public health, as climate change and urbanization expand the range of vector-borne diseases like West Nile virus, dengue, chikungunya, and Zika. Studies from regions such as Southern Europe and the Middle East underscore the need for enhanced monitoring systems in Iran. The detection of *Culex* species in Iran parallels findings from Europe, highlighting similar risks. Advances in molecular techniques, particularly real-time PCR, have improved virus detection, supporting the choice of methodologies in surveillance research. Comparisons with similar ecological regions can contextualize the study’s findings and address the influence of mosquito species diversity on arbovirus transmission. Additionally, citing regional studies justifies the necessity for surveillance in Iran, reflecting broader trends in neighboring countries.

The manuscript addresses critical gaps in arbovirus surveillance in the Middle East and North Africa, stressing the importance of the current research. The manuscript is encouraged to propose future research directions, such as expanding surveillance and using climate models for vector population predictions.

The detection of *C. tarsalis* in Iran carries significant implications for public health, particularly concerning the potential transmission of arboviruses like West Nile virus and other pathogens. This finding underscores the necessity for enhanced surveillance and vector control measures in response to the evolving landscape of mosquito-borne diseases. Additionally, the identification of new mosquito species emphasizes the importance of research and collaboration in understanding vector ecology and mitigating public health risks.

## Materials and methods

### Study area

**Figure 1.**
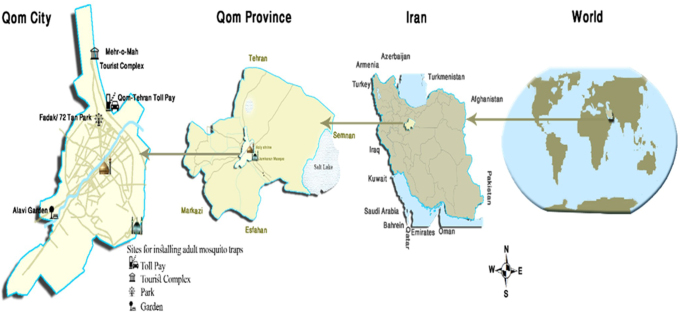
Qom province in Iran, the study map.

The Qom province in Iran (Fig. 1) is located in the center of the country with an altitude of 900 m above sea level. The population is approximately 1 365 000, and different types of climatic conditions are seen such as the mountainous in the western and southern parts, semi-desert in the central part, and hot and dry desert in the Eastern regions. The warmest months are June and July, while January is the coldest month.

### Collection of Culicidae mosquitoes

**Figure 2.**
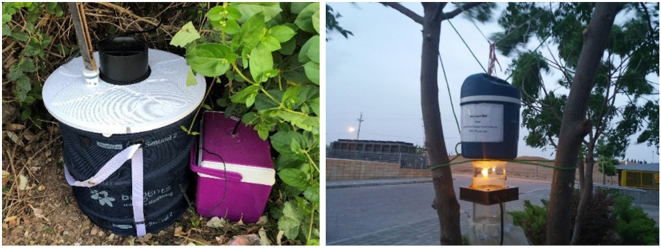
*Left*: Biogents (BG) trap and *Right*: CO_2_ trap.

**Figure 3.**
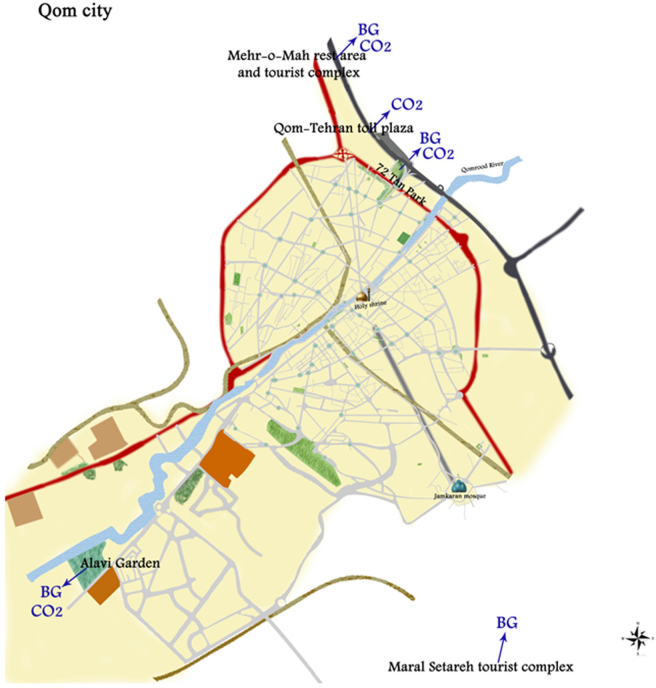
Map of the study area, Qom city, Qom province, Iran, 2018.

Eight traps (four Biogents (BG) traps and four CO_2_ traps) were used to catch adult mosquitoes (Fig. 2). Five separate ecological zones were identified for the field placement of eight traps in the Qom province. The traps were placed at the entrances of the city of Qom, from the north, northwest, southeast, and southwest of the country. One CO_2_ trap and one BG trap were placed in the Mehr-o-Mah rest area and tourist complex, one CO_2_ trap was placed in the Qom-Tehran toll plaza, one CO_2_ trap and one BG trap were placed in the 72 Tan Park, one BG trap was placed in the Maral Setareh tourist complex, and one CO_2_ trap and one BG trap were placed in the Alavi Garden (Fig. 3).

Mosquitoes contribute significantly to local ecosystems, serving as food sources for predators and as pollinators, indicating ecological health. This study identified 11 mosquito species, with their diversity reflecting a balanced ecosystem. As environmental indicators, certain species can signal habitat quality changes, while the presence of diverse species suggests potential risks for disease transmission, even when not directly linked to outbreaks. Key species like *C. tarsalis* are known vectors for West Nile virus, while *Aedes* species may carry other arboviruses. Competition for resources among species can influence population dynamics and disease risk, highlighting the necessity for ongoing monitoring to understand shifts in transmission patterns due to ecological changes.

The diversity of 11 mosquito species in a study raises important questions about vector competence related to virus transmission. Different species may serve varying roles as primary, secondary, or incidental vectors, increasing co-infection risks and complicating disease dynamics. This highlights the necessity for targeted vector control strategies tailored to the ecological and behavioral characteristics of each species, as well as the implementation of integrated vector management approaches. Effective control measures should include habitat reduction, insecticide use, and public education. Additionally, comprehensive mosquito monitoring programs are essential for tracking population changes and mitigating arbovirus outbreak risks in response to environmental factors. The study emphasizes the ecological importance of mosquito diversity and the need for ongoing surveillance and targeted strategies.

The BG traps were installed before sunrise. The CO_2_ traps were installed at dusk and dismounted half an hour after sunrise of the following day. In CO_2_ traps, light was used as an additional adult mosquito’s attractant. Traps were installed in public places, such as parks and tourist complexes. The *Culicidae* mosquitoes were morphologically identified within 1–2 h, and some of them were selected as pool and kept at liquid nitrogen for further investigation for species of mosquitoes and possible presence of arboviral infection.

### Morphological identification of mosquitoes

Collected mosquitoes were transferred to the entomology laboratory and placed in dry ice for a few minutes, and microscopic diagnosis was done within 1–2 h, as previously described^[22,23]^. In microscopic diagnosis, diagnostic keys and a 60× stereomicroscope lens were used. Undetectable or suspected mosquitoes and a number of identified mosquitoes were selected for molecular testing. Each pool contained an average of 40 mosquitoes of one species; of some species, only one mosquito was caught.

### Molecular identification of mosquitoes

A total of 20 pools of mosquitoes were subjected to molecular identification and morphological identification. These 20 pools were selected randomly and sent to the Department of Arboviruses and Viral Hemorrhagic Fevers (National Ref Lab), Pasteur Institute of Iran, while maintaining the cold chain using dry ice and a cold box for molecular investigations. All samples were stored at −70°C immediately after receiving for further analyses.

The rationale for selecting only 20 pools can be further expanded. Although logistical limitations impacted the sample size, we strategically selected pools to cover diverse species and geographic zones, thereby maximizing representativeness across the mosquito population in Qom. This selection aimed to ensure that the chosen pools accurately reflected the ecological and virological characteristics of the entire sample.

The selection of 20 pools for molecular analysis was driven by logistical constraints, resource availability, and the need for scientific rigor. Molecular analysis requires specialized resources and is time-consuming, making it impractical to test all samples. To ensure validity, the pools were randomly selected to represent the diversity and geographic range of mosquito populations. This strategy included a variety of species from different ecological settings, covering urban and rural areas, which enhances the reliability of the findings regarding arbovirus transmission risk. Despite the limited sample size, the approach aimed to provide a comprehensive assessment of the broader mosquito population.

Mosquito samples were preserved at −80°C for RNA integrity before extraction for molecular analyses targeting arboviruses, particularly West Nile virus. Total RNA was reverse transcribed into cDNA using a high-fidelity enzyme, followed by amplification of specific arboviral genes through Reverse transcription polymerase chain reaction (RT-PCR). Challenges included RNA degradation, which was mitigated by using RNase-free reagents and controlled conditions; however, some degradation impacted cDNA quality. Sensitivity issues were addressed by including control samples, and PCR conditions were optimized to enhance specificity. Future research could utilize multiplex PCR and advanced techniques like quantitative PCR and next-generation sequencing for improved detection and understanding of arboviruses.

Future research should prioritize the screening of arboviruses with significant public health implications, specifically West Nile virus, St. Louis Encephalitis virus, chikungunya virus, dengue virus, and Zika virus. Surveillance for these viruses is critical due to their potential for outbreaks and associated health risks. Integration of climate models is also important to predict changes in mosquito distribution and transmission dynamics as influenced by climate change. Collaborative efforts among neighboring countries, including establishing surveillance networks and standardized protocols, are essential for improving detection and response to arboviral threats.

For molecular characterization of mosquitoes, PCR amplification and sequence analysis of 735 base pair fragments, including mitochondrial COI gene was carried out using SP ID F/SP ID R primers^[24]^. Additionally, the specific primers for actin-1 (ActF and R)^[25]^ were used. Briefly, mosquitoes were homogenized using Tissuelyser II (Qiagen, Germany). The genomic DNA was extracted from pools using a High Pure Viral Nucleic Acid Kit (Roche, Germany) according to the manufacturer’s instructions. PCR was performed by the Hot StarTaq Master Mix Kit (Qiagen, Germany).

The final volume was 20 μL for each reaction, performing one cycle at 95°C for 15 min, five cycles at 94°C for 40 s, 45°C for 60 s, and 72°C for 60 s, and 35 cycles at 94°C for 40 s, 51°C for 60 s, and 72°C for 60 s, and a final extension at 72°C for 10 min. PCR products (735bp for SP ID primers and 350bp for ACT primers) were sequenced bidirectional using applied biosystem 3130 Genetic Analyzer (Thermofisher, USA). The sequencing raw data were analyzed by CLC Main Workbench software (CLC bio, Denmark) and using BLAST (https://blast.ncbi.nlm.nih.gov/Blast.cgi) for confirmation, then all sequences data were submitted to GenBank (https://www.ncbi.nlm.nih.gov/genbank) database (Table 1).

**Table 1:** Evaluation of mosquito detection by microscopic method and confirmation by Sanger sequencing

No.	Possible microscopic detection of species	The list of mosquitoes after sequencing and blasting	Accession number	Results
1	*Culex pipiens*	*Culex pipiens*	OM714817	Compatible
2	*Culex pipiens*	*Culex pipiens*	OM628998	Compatible
3	***Culex*** spp.	***Culex tarsalis***	ON420215	Incompatible
4	*Culex theileri*	*Culex theiler*	OM638648	Compatible
5	*Culex modestus*	*Culex modestus*	OM638670	Compatible
6	*Culex subochrea*	*Culex subochrea*	Not sequenced	-
7	*Culex perexiguus*	*Culex perexiguus*	Not sequenced	-
8	*Anopheles*	*Anopheles claviger*	OM638671	Compatible
9	*Aedes caspius*	*Ochlerotatus caspius*	OM638672	Compatible
10	*Aedes caspius*	*Ochlerotatus caspius*	OM638673	Compatible
11	*Aedes caspius*	*Ochlerotatus caspius*	OM638674	Compatible
12	*Aedes caspius*	*Ochlerotatus caspius*	Not sequenced	-
13	*Culex* spp.	*Culex theilerivoucher*	OM638676	Incompatible
14	*Culiseta longiareolata*	*Culiseta longiareolata*	OM638715	Compatible
15	*Culex quinquefasciatus*	*Culex quinquefasciatus*	OM638717	Compatible
16	*Culex* spp.	*Culex quinquefasciatus*	OM714908	Incompatible
17	*Culex quinquefasciatus*	*Culex quinquefasciatus*	OM714856	Compatible
18	*Culex quinquefasciatus*	*Culex quinquefasciatus*	OM714858	Compatible
19	*Culex quinquefasciatus*	*Culex quinquefasciatus*	OM714899	Compatible
20	*Culex quinquefasciatus*	*Culex quinquefasciatus*	Not sequenced	-

Bold entries indicate discrepancies in species identification between morphological examination and molecular sequencing.

### Detection of arboviruses

Homogenized mosquitoes were used for total RNA extraction using High pure viral RNA kit (Roche, Germany). The presence of Alphaviruses, Flaviviruses, and Phleboviruses RNAs was investigated by Pan Phlebo RT-PCR using ppLF/ppL1R/ppL2R primes, Pan Flavi RT-PCR using MAMD/cFD2 primers, and Pan Alpha RT-PCR using E1-S/E1-C primers^[26]^. All RT-PCR reactions were done using Onestep RT-PCR kit (Qiagen, Germany). The final volume for each reaction was 25 μL performing one cycle at 50°C for 30 min, 95°C for 15 min, 40 cycles at 94°C for 30 s, 53°C for 30 s, and 72°C for 45 s, and a final extension at 72°C for 5 min.

## Results

In this study, during 2019–2020, a total of 83 414 mosquitoes were caught. According to the morphological key, all categorized as *Culicidae* addition 933 Sandfly were caught. Of which, 27,057 *Culicidae* and 116 Sandfly were selected and kept at liquid nitrogen in 367 Pool including 355 *Culicidae* mosquitoes, and 12 were *phlebotomine*. Since the microscopic and molecular diagnoses of *phlebotomine* were the same and no arboviruses were discovered in them, in this article, we will not continue the discussion about phlebotomines and *Culicidae*, each pool contained an average of 76 mosquitoes of one species. To evaluate the results of morphological identification, 20 pools were subjected to morphological identification, as well.

The results of morphological and molecular characterization of mosquitoes were incompatible for three pools (Table 1). Pools no. 3, no. 13, no. 16 were categorized as just *Culex* because their species could not be recognized. In morphological identification, the pool no. 3 was characterized as *C. tarsalis* after genomic analysis. According to the molecular assay, the pool no. 13 was *Culex theilerivoucher*, and the pool no.16 was categorized as *Culex quinquefasciatus* in the molecular assay.

The negative findings regarding arboviruses should be interpreted as a valuable part of understanding the local vector ecology and public health risks. These results underscore the importance of sustained, comprehensive surveillance to detect future changes in virus circulation. By addressing potential reasons for the negative results—such as seasonal factors, assay sensitivity, and low virus circulation—the study provides a strong foundation for improving future surveillance and vector control programs in the region.

**Figure 4.**
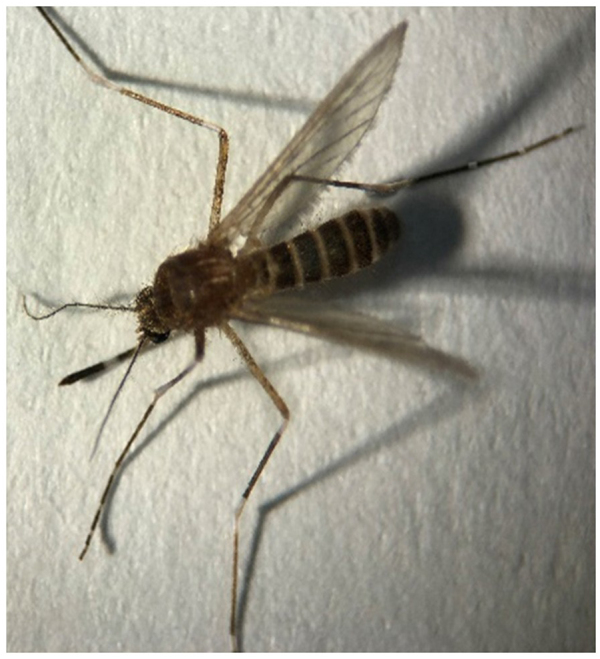
*Culex tarsalis. Cx. tarsalis* was not included in the diagnostic keys of Iran. The genus was identified by the entomologist, while species identification was confirmed by the national reference laboratory.

**Figure 5.**
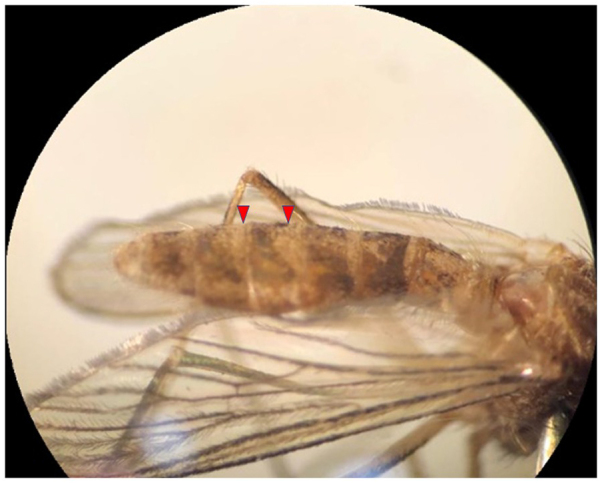
*Cx. theileri* (unknown to Entomologist).

In comparison, in the microscopic method, of these 20 Pools, three were misdiagnosed or incorrect. *C. tarsalis* has been reported for the first time in Iran, and the main reason for the lack of detection by the entomologist was the absence of this mosquito in the available diagnostic keys in Iran. Therefore, the entomologist could only determine the genus (Fig. 3). The *Culex theileri* has a bright transverse band on the abdomen^[27,28]^, but in our study, it was a rectangular band in the third abdominal band (Figs. 4 and 5) (Table 1). No Alphavirus, Flavivirus, or Phlebovirus infection was identified in the collected mosquitoes.

**Figure 6.**
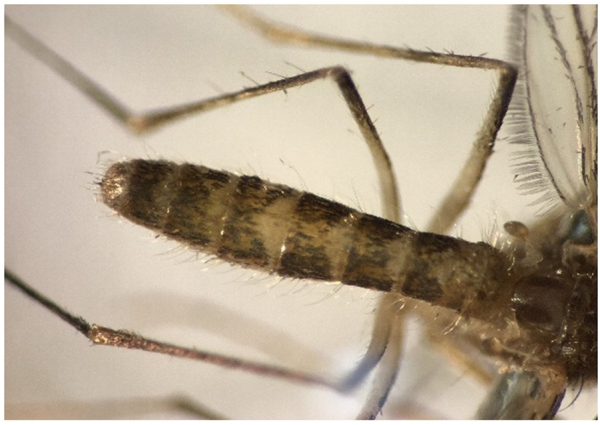
*Cx. theileri*.

*C. theileri* has a bright transverse band on the abdomen. There is a rectangular band in the third abdominal band. Of course, it has the vertex of a bright triangle (Fig. 6).

## Discussion

Considering that Qom province is a central city for the transit of goods to the south and north of Iran, mosquitoes can be moved mechanically into or out of this region. In addition, a large number of tourists from Iran or other Muslim countries travel to this city for religious purposes and can be bitten by mosquitoes during those periods of residency.

In our study, 11 species were collected and identified, which included *C. quinquefasciatus, Culex pipiens, C. theileri, Culex subochrea, Aedes (Ochlerotatus) caspius, Culex modestus, C. theileri voucher, Anopheles claviger, Culex perexiguus, Culiseta longiareolata,* and *C. tarsalis* in Qom province. *C. tarsalis* was identified for the first time from Iran. Asgarian *et al*. in fauna and larval habitat, from April to December 2019, identified three genera *Anopheles, Culiseta*, and *Culex* in Kashan and also *Culex pipiens* (37.36%) and *Cx. Theileri* (26.10%) had the highest abundance of species^[15]^. Nikookar *et al*. reported that *C. pipiens* as the most abundant species in fauna and larval habitat in Neka County, Northern Iran^[29]^.

Moradi-asl *et al*. identified 13 species of mosquitoes in the form of four genera (Anopheles, Aedes, Culex, and Culiseta) in the north-western province of Iran, and in this study, the Culex and *Culex pipiens* were the most abundant genus and species, respectively^[30]^.

In the current study, the most identified species is *C. quinquefasciatus*, but Saghafipour *et al*. in Qom province reported four genera among Diptera: *Culicidae*, which included 14 species, and from them, *anopheles claviger* was the most common species^[31]^. Therefore, it seems that the identification of different rates among the species can be due to different conditions and climatic variables. On the other hand, morphological studies alone are not sufficient to differentiate species, and molecular methods can be helpful in identifying the species with high sensitivity. *C. tarsalis* are strong flyers, covering distances of up to 4 km for a blood meal and feeds from birds, cattle, horses, and human^[32]^.

Finding a new species of mosquito for the first time in Iran also warns that we must always wait for new species of mosquitoes, their establishment, and the potential spread of emerging and re-emerging diseases. In addition, because of the genetic and behavioral changes in each species, physical diagnosis becomes more difficult, and therefore molecular detection should always be used besides physical and morphological diagnosis.

Except for some molecular and serological reports for West Nile virus in Iran, chikungunya virus and dengue viruses have been reported as imported cases into Iran. In the present study, no Alphavirus, Flavivirus, and Phlebovirus have been reported^[10,24,33-35]^.

However, due to climate change, there is a possibility that these viruses will spread in the future. Therefore, continuous entomological and virological studies, along with field studies on vectors in the region, seem to be necessary.

## Conclusion

The weather and climate changes that have occurred in Iran have caused the appearance of new species and even changes in the lifestyle of mosquitoes. Therefore, continuous studies in different geographical areas on viral vectors provide new information about the spread of arboviral diseases and their vectors.

## Data Availability

All study data are included in the text of the article and available.
